# Anatomical description of the brain wax models of Museu da Pharmacia de Ouro Preto

**DOI:** 10.1055/s-0042-1758463

**Published:** 2022-12-28

**Authors:** Luiz Eduardo Sousa, Ingrid da Silva Borges, Helen Seidel, Isadora Lopes Pereira, Juliana de Paula Farias

**Affiliations:** 1 Universidade Federal de Ouro Preto, Departamento de Ciências Biológicas, Laboratório de Anatomia Humana, Ouro Preto MG, Brazil.; 2 Universidade Federal de Ouro Preto, Museu da Pharmacia, Ouro Preto MG, Brazil.; 3 Universidade Federal de Ouro Preto, Escola de Medicina, Ouro Preto MG, Brazil.; 4 Universidade Federal de Ouro Preto, Departamento de Museologia, Ouro Preto MG, Brazil.

**Keywords:** Models, Anatomic, Neuroanatomy, Education, History, Modelos Anatômicos, Neuroanatomia, Educação, História

## Abstract

**Background** In 1839, the Escola de Farmácia de Ouro Preto pioneered the teaching of pharmaceutical sciences in Brazil. At the end of the 19th century, the Escola de Farmácia possessed a French collection of anatomical models, some made of wax and *paper-mâché* . The models were a critical part of teaching anatomy, particularly in an era of paradigm changes about how the human brain works.

**Objective** The present study aimed to anatomically describe the brain models through a comparative analysis with the current anatomical description.

**Methods** Comparative analysis of the brain models with modern anatomical descriptions.

**Results** In the individual analysis of the wax models, we verified excellent anatomical accuracy of the cortical and subcortical regions. Our results identified internal structures, like the basal ganglia and white matter. Compared with modern anatomical books and websites, the wax brain models have high scientific quality.

**Conclusion** The models of the present study gave students hands-on experience of human anatomy in the 19 ^th^ century. Nowadays, the models are part of the memory of Universidade Federal de Ouro Preto and Museu da Pharmacia de Ouro Preto . The collection of wax models shows the appreciation of neuroanatomy teaching at the turn of the century concomitant with advances in neurology and anatomy around the world.

## Resumo

**Antecedentes** No Brasil, em 1839, a Escola de Farmácia foi pioneira no ensino das ciências farmacêuticas. No final do século XIX, a Escola de Farmácia possuía uma coleção de modelos anatômicos franceses, muitos fabricados em cera e papel machê. A coleção da Escola de Farmácia é muito importante pois faz parte de um período crítico do ensino de neuroanatomia, particularmente em um período de mudanças de paradigmas relacionados às funções do cérebro humano.

**Objetivo** Os objetivos do presente estudo foram analisar anatomicamente os modelos de cérebro e identificar e descrever as estruturas anatômicas com base na descrição anatômica moderna.

**Métodos** Foi feita uma análise comparativa dos modelos com a descrição anatômica moderna.

**Resultados** Na análise individual dos modelos de cera, nós constatamos uma excelente fidelidade anatômica de regiões corticais e subcorticais. Nossos resultados identificaram estruturas internas, como núcleos cerebrais e substância branca. Comparado com livros e páginas da internet sobre anatomia, os modelos de cera apresentam alta qualidade científica.

**Conclusão** Os modelos do presente estudo são importantes, pois ajudam a compreender o ensino de anatomia no século XIX. Atualmente, os modelos fazem parte da memória da Escola de Farmácia de Ouro Preto e do Museu da Pharmacia. A coleção de modelos de cera representa a valorização do ensino de neuroanatomia no final do século XIX em concordância com os avanços da neurologia e neuroanatomia ao redor do mundo.

## INTRODUCTION

In Europe, between the 17 ^th^ and 19 ^th^ centuries, the development of models of teaching in human anatomy increased due to the scarcity of corpses in medical schools, the great difficulty in preserving bodies, and the need to create durable models for teaching. 1 The first teaching wax model was made by the Sicilian Gaetano Giuliano Zumbo in the 17 ^th^ century. 2 After the 18 ^th^ century, anatomical models were already widely used. 2

In Brazil, Escola de Farmácia de Ouro Preto pioneered the teaching of pharmaceutical sciences. Founded in 1839, the Escola de Farmácia de Ouro Preto, in Minas Gerais, introduced a pharmacy specialty without fellowship to the medical school. 3 At the end of the 19th century, Escola de Farmácia de Ouro Preto had a French collection of anatomical models, some made of wax and *paper-mâché* . 4 This collection is currently in Museu da Pharmacia (the Pharmacy Museum), in the historic centre of Ouro Preto, Brazil. 4 In the 1960s, materials were acquired for the formation of Museu da Pharmacia, offering visitors an exhibition of the equipment used in the training of pharmacists and their workplace. The museum has a collection on the teaching and professional practice of pharmacy, with medicines and equipment in chemistry, physics, human anatomy, and biology. 3 The Escola de Farmácia de Ouro Preto collection is very important, as these anatomical models were a critical part of teaching anatomy in Brazil, particularly in an era of paradigm changes about the function of the human brain.

The models of the present study were used in the anatomy laboratory of Universidade Federal de Ouro Preto until 2018. Since then, the models have been retrieved and transferred to Museu da Pharmacia.

In 19 ^th^ century Europe, renowned manufacturers, such as the French Deyrolle, Dr Auzoux, and the Vasseur-Tramond workshop, made anatomical models with high scientific quality. However, there is no data regarding the anatomical analysis of wax brain models from the 19th century provided by Paul Rousseau & Cie., Paris - France. According to data from the Pharmacy Museum, Paul Rousseau & Cie. was not a manufacturer (because the manufacturer is unknown), but a scientific equipment store in Paris that supplied Escola de Farmácia de Ouro Preto. Thus, the present study aimed to anatomically describe the brain models through a comparative analysis with the current anatomical descriptions.

## METHODS

**Figure 1.**
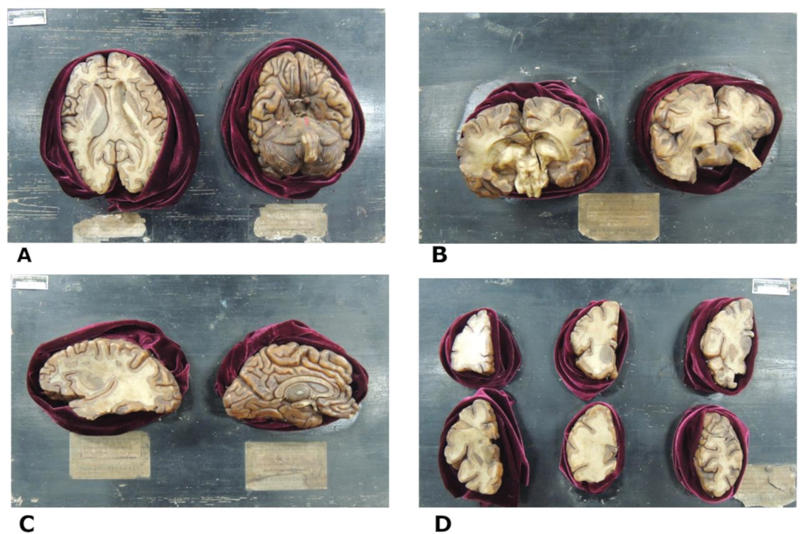
The anatomical wax models are attached to labelled wooden bases. Base A – Cross section of the cerebral hemispheres (left) and inferior view of the brain (right); Base B – Two coronal sections of the brain; Base C – Sagittal section of the cerebral hemisphere (left) and medial view of the left cerebral hemisphere (right); Base D – Six coronal sections of the frontal lobe of the brain.

Four bases containing 12 French wax brains provided by Paul Rousseau & Cie. Produits Chimiques Ustensiles de Laboratoire – Instruments de Physique Matériel Scolaires, Paris – France, were analyzed ( Figure 1 ). Base A had two models: a brain in cross-section and a lower view of the brain. Base B had two models of the brain in coronal sections. Base C had a sagittally-sectioned hemisphere and a medial view of the left cerebral hemisphere. Base D had six small models of the frontal lobe in coronal section.

The materials were photographed with a Nikon P510 camera on a tripod. All pictures were taken at the studio of photography at the Pharmacy Museum of the Universidade Federal de Ouro Preto. The photo table consisted of four fluorescent lights around the backdrops. The distance and position of the lights were adjusted to minimize the reflection in the wax. The photographs were taken perpendicularly to the plane of the objects, at the same distance. The software GIMP 2.10.2 was used for editing images. The cracks and broken parts of the models were identified in the pictures with a dotted white line. Neuroanatomic structures were identified with a black and white line. A black background was added to improve the contrast and the identification of the anatomic structures.

A comparison of model pictures with images from books and a neuroanatomy website was performed to determine anatomical accuracy. A qualitative analysis of the wax models was carried out, which consisted of the identification of brain regions and anatomical variations, in comparison with current bibliographic references. The accuracy was determined by the similarities between the brain regions and the current bibliography. The shape of the anatomical region, location, anatomical relationships, and proportion were analyzed. The models were analyzed by a professor anatomist. The reference books were Functional Neuroanatomy and Atlas of Human Anatomy. 5 6 The reference website consulted was *Atlas of the Human Brain* . 7 No identifications were made on the damaged areas of the wax models. In models with bilateral symmetry, the identification was made on the side with better quality.

A comparative analysis was performed between the wax model and the *Atlas of the Human Brain* . 7 For the analysis of the wax models that represent the inferior and medial views, images from the Atlas of the Human Anatomy and Functional Neuroanatomy were used. 5 6

## RESULTS

**Figure 2.**
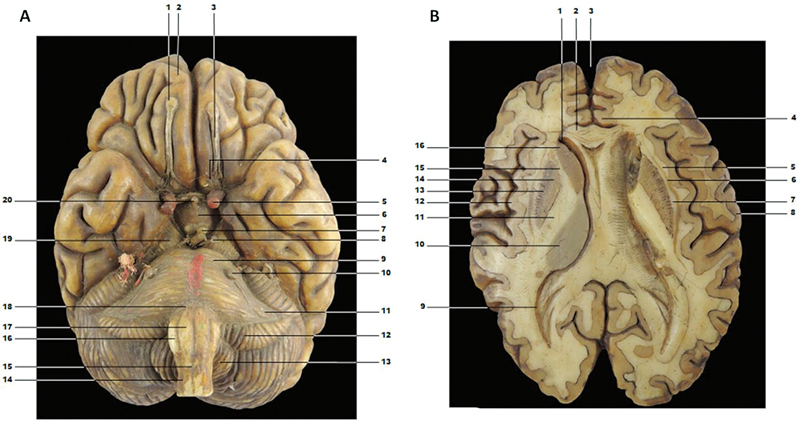
A - Wax model in lower view of brain, brainstem, and cerebellum. 1 - olfactory bulb; 2 - frontal lobe; 3 - olfactory tract; 4 - medial and lateral olfactory stria; 5 - internal carotid artery; 6 - tuber cinereum; 7 - mammillary body; 8 - oculomotor nerve; 9 - pons; 10 - trigeminal nerve; 11 - middle cerebellar peduncle; 12 - cerebellum flocculation; 13 - cerebellar tonsil; 14 - anterior lateral sulcus of the bulb; 15 - anterior median fissure of the bulb; 16 - olive; 17 - pyramid; 18 - pontomedullary junction; 19 - cerebral peduncle; 20 - hypophysis. B - Wax brain cross section. 1 - anterior horn of lateral ventricle; 2 - corpus callosum; 3 - longitudinal fissure; 4 - cingulate gyrus; 5 - extreme capsule; 6- claustrum; 7 - external capsule; 8 - postcentral gyrus; 9 - posterior horn of the lateral ventricle; 10 - thalamus; 11 - internal capsule; 12 - insular cortex; 13 - putamen; 14 - globus pallidus; 15 - internal capsule; 16 - head of caudate nucleus.

In the individual analysis of the wax models, we verified excellent anatomical fidelity of cortical and subcortical regions. Figure 2B represents a horizontal section of the brain with a clear distinction between the cerebral cortex and white matter. The color differentiation between the cortex and white matter exists in other models. The claustrum, putamen, and globus pallidus were manufactured in a dark color. Despite the correct representation of many anatomical regions, the model in Figure 2B has a slight asymmetry between the hemispheres. We observed a slight anatomical difference between the lateral ventricles, particularly in the posterior horn.

Figure 2A represents the lower surface of the brain, including the gyri, sulci, brainstem (midbrain, pons, and medulla), and cerebellum. In this model, the olfactory bulb, olfactory tract, oculomotor nerve, and trigeminal nerve were the structures associated with the cranial nerves, and the internal carotid arteries represented vascular structures. Wax cerebellum modelling is similar to modern anatomical description, as found in the cerebellar hemispheres, flocculus, and cerebellar tonsil. Damaged regions affected the oculomotor nerves into the interpeduncular fossa. We also found no representation of the other cranial nerves, as can be seen on the surface of the brainstem.

**Figure 3.**
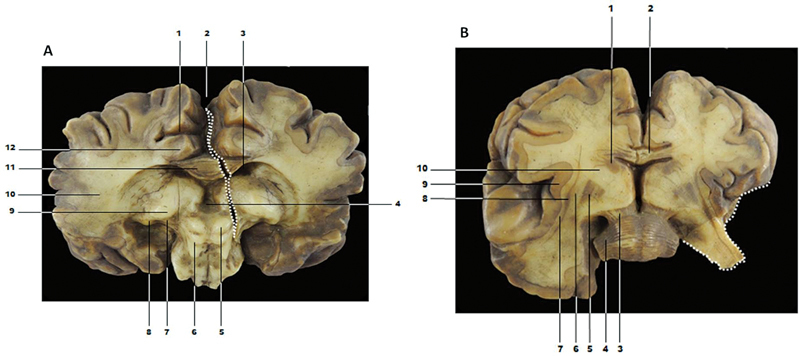
A – Coronal section of the brain and brainstem - posterior view. The damage area is defined by the dotted white line. 1 - cingulate sulcus; 2 - longitudinal fissure; 3 - right lateral ventricle; 4 - third ventricle; 5- right colliculus superior; 6 - left colliculus inferior; 7 - medial geniculate body; 8- lateral geniculate body; 9 - thalamus; 10 - telencephalon white matter; 11 - corpus callosum; 12 - cingulate gyrus. B – Coronal section of the brain and brainstem - anterior view. The damage area is defined by the dotted white line. 1 - head of caudate nucleus; 2 - corpus callosum; 3 - cerebral peduncle; 4 - middle cerebellar peduncle; 5 - lentiform nucleus; 6 - external capsule; 7 - extrema capsule; 8 - claustrum; 9 - insular cortex; 10 - internal capsule.

The wax models in the present study are parts of the brain in different perspectives and sections. Figure 3 shows a coronal section of the brain and brainstem in a posterior and anterior view. We identified the following brain regions in the model: the thalamus, corpus callosum, third ventricle, colliculus, and geniculate bodies ( Figure 3A ). A crack was identified in the center of the model, but it did not affect the anatomical identification.

Figure 3B is an anterior view of the coronal section of the brain. In this wax model, we identified the basal ganglia, brain cortex, corpus callosum, and pons. A part of the model was broken (white dotted line), which made it impossible to identify structures in these regions.

**Figure 4.**
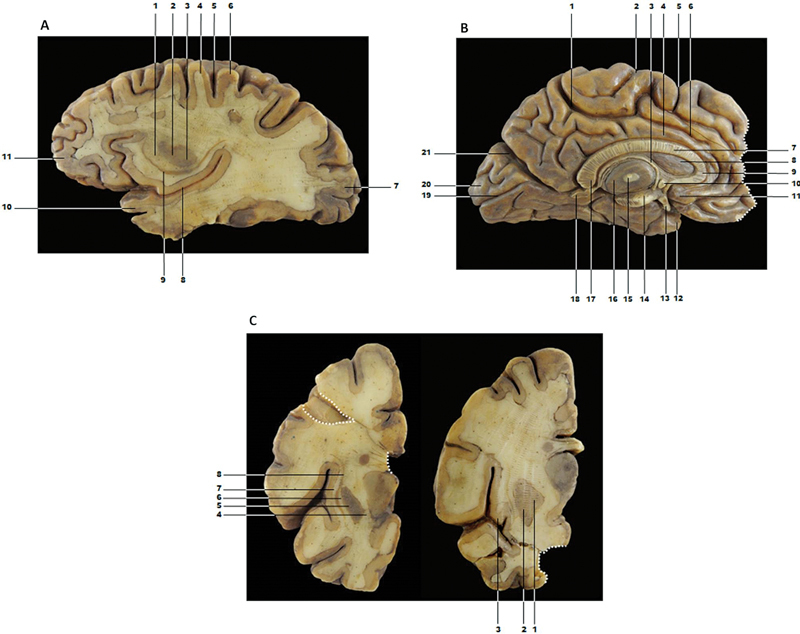
A – Parasagittal section of the brain. 1 - internal capsule; 2 - putamen; 3 - globus pallidus; 4 - precentral gyrus; 5 - central sulcus; 6 - postcentral gyrus; 7 - occipital lobe; 8 - lateral sulcus; 9 - claustrum; 10 - upper temporal gyrus; 11 - frontal lobe. B – Medial view of the left cerebral hemisphere. The damage area is defined by the dotted white line. 1 - marginal branch of cingulate sulcus; 2 - central sulcus; 3- fornix body; 4 - cingulate gyrus; 5 - paracentral sulcus; 6 - cingulate sulcus; 7 - trunk of corpus callosum; 8- septum pellucidum; 9 - genu of corpus callosum; 10 - anterior commissure; 11 - septal area; 12 - uncus; 13 - pituitary stalk; 14 - cerebral peduncle; 15 - interthalamic adhesion; 16 - thalamus; 17- splenium of corpus callosum; 18 - pineal body; 19 - calcarine sulcus; 20 - cuneus; 21 - parieto-occipital sulcus. C – Two coronal section of the frontal lobe. The damage area is defined by the dotted white line. 1 - globus pallidus; 2 - putamen; 3 - insular cortex; 4 - internal capsule; 5 - putamen; 6 - external capsule; 7 - extreme capsule; 8 - claustrum.

The wax brain collection has typical brain sections but includes an unusual parasagittal section ( Figure 4A ). This parasagittal section shows the basal ganglia, as well as the cortex.

Figure 4B shows the medial face of the cerebral hemisphere with the following regions highlighted: cortical areas, the diencephalon, gyri, and sulci, such as the cingulate sulcus, calcarine sulcus, central sulcus, cingulate gyrus, septal area, thalamus, and pineal body. In this model the frontal lobe was broken, making it impossible to identify anatomical structures of the frontal pole. The models in Figure 4C show important internal regions of the brain, such as the claustrum, putamen, globus pallidus, extreme capsule, and insular cortex.

Base D ( Figure 1 ) has six wax models of the frontal lobe of the brain; however, two models had damage that impaired anatomical identification. Two models were removed from the study, as the exact section's location was not identifiable.

All bases of the wax models had original French labels. Unfortunately, the labels had deteriorated due to conditions that the models have been exposed to over the years.

## DISCUSSION

The wax models examined in this study portray an important chapter in the history of anatomy, especially for the pharmaceutical sciences of Brazil, in addition to their scientific relevance. Despite the similarity with the models of Vasseur-Tramond (Anatomical Museum of the University of Valladolid, Spain), we cannot certify the manufacturer.

In France, famous anatomists and companies innovated and produced fabulous wax models of the human brain anatomy. 8 9 10 The Vasseur-Tramond workshop created a studio of ceroplasty nearby the Paris anatomy amphitheater, in the 19 ^th^ century. Classical wax models were created, such as models of the ears, heart, and brain. Among the most famous models, the Vasseur-Tramond workshop made a head that showed the cranial nerves, muscles, tongue, and bones. 8 The realism of the wax models is due to the creators' experience, skill, and anatomical knowledge. It is assumed that manufacturing of the model required two specialists: an anatomist doctor for dissection and a sculptor to reproduce it in wax. 9 11 Our models have good differentiation between white (internal capsule, for example) and gray matter (putamen, for example). The quality of the models was improved by mixing materials in the wax to provide different textures and colors. 9 11

Our results show anatomical accuracy between the brain models compared to anatomical descriptions in current books. 5 12 13 Regions of the cerebral cortex, such as the central sulcus, lateral sulcus, precentral gyrus, postcentral gyrus, cingulate gyrus, and insular cortex, were represented according to the modern anatomical description. 5 When analyzing the internal anatomy of the wax brain models, we found an anatomical equivalence of the thalamus, basal ganglia, and white matter of the brain, such as in the internal capsule, globus pallidus, putamen, and external capsule. Thus, we found that the models of the present study have an excellent anatomical accuracy when compared to the descriptions of current neuroanatomy textbooks. 5 7

In the brain section of Figure 2B , we found a different anatomical representation of the lateral ventricle. We supposed that this inconsistency of the lateral ventricle was due to technical difficulties in modelling this region, in spite of the advanced knowledge on the anatomy of the ventricles in the 19 ^th^ century. Historically, the shape of the lateral ventricle was studied by Leonardo da Vinci (1506–1508), who injected hot wax directly into the brain of an ox. After the wax cooled, Da Vinci dissected away the brain tissue to produce a cast of the brain ventricles. 14

The models in our study are from an important era of discoveries in neuroanatomy. The interest in the study of the human brain increased in the 19 ^th^ century, mainly through surgical experiments. This scientific advancement was reflected in the increased demand for materials for practical classes on human anatomy at European universities. Thus, the use of brain wax models allowed the study of the brain in medical schools in a period of increasing scientific interest in the nervous system.

In the 19 ^th^ century, important studies broke the paradigm in neuroanatomy, which began placing anatomical knowledge in the modern age of science. In this time, Franz Joseph Gall was considered the pioneer of the theory of cortical location in the brain and phrenology. 15 Other pioneers were Paul Broca (1824–1880), who located brain regions associated with language and speech, and Richard Caton, who was likely the first to record brain electrical activity. 15 In the late 19 ^th^ and early 20 ^th^ centuries, research was conducted on the cytoarchitecture of the cerebral cortex by Oscar Vogt, Cécile Vogt, Grafton Elliot Smith, Alfred Walker Campbell, and Korbinian. 15 These and other researchers have changed the course of knowledge regarding the human brain. With attention focused on the anatomy of the brain, medical schools likely needed more anatomical teaching models.

When compared to modern teaching models, wax models are considered fragile, especially when exposed to heat. However, when properly preserved, the wax is very stable and durable. 16 There are many anatomy museums around the world with historical wax models, mainly in Europe, such as Museum ‘La Specola’ (Florence, Italy) and King's College London School of Medicine (London, UK). In Brazil, the Museu de Anatomia Humana Professor Alfonso Bovero (São Paulo, Brazil) and the recently opened Anatomy Exhibition of Museu da Pharmacia (Ouro Preto, Brazil) have a collection of historical 19 ^th^ century models.

Our results show that Escola de Farmácia de Ouro Preto, prized for excellence in teaching human anatomy, has an excellent collection of anatomical models, including the collection from Vasseur-Tramond, Dr Auzoux, and Deyrolle. 3 17 However, the wax brain model manufacturer is unknown. Indeed, the exhibition of human anatomy at Museu da Pharmacia contributes to the education of high school students and offers a cultural experience regarding the history of anatomy and pharmacology in Brazil. Museu da Pharmacia also exhibits many pieces of pharmacology equipment, old drugs, books, pictures, and other items related to the early years of Escola de Farmácia de Ouro Preto.

The collection shows excellent accuracy, and all anatomical structures were compatible with the current bibliography. The models of the present study gave students hands-on experience of human anatomy in the 19 ^th^ century. Nowadays, the models are part of the memory of Escola de Farmácia de Ouro Preto and Museu da Pharmacia. The collection of wax models shows the appreciation of neuroanatomy teaching at the turn of the century concomitant with advances in neurology and anatomy around the world.
